# Formulation Development of Fluconazole-Loaded Lactose Agglomerate Tablets as a Disinfectant for *Candida*-Associated Dentures

**DOI:** 10.3390/pharmaceutics14081723

**Published:** 2022-08-18

**Authors:** Rapee Jarungsirawat, Wanassnant Kajthunyakarn, Chaipat Siriwachirachai, Thaned Pongjanyakul

**Affiliations:** 1 Division of Pharmaceutical Technology, Faculty of Pharmaceutical Sciences, Khon Kaen University, Khon Kaen 40002, Thailand; 2 Department of Pharmaceutical Technology, Faculty of Pharmacy, Srinakharinwirot University, Nakhon Nayok 26120, Thailand

**Keywords:** fluconazole, denture, anticandidal, *Candida albicans*, lactose, tablets, agglomeration

## Abstract

Denture stomatitis is induced by irritation or an inflammatory response when wearing a denture for a long time. *Candida* species are the leading cause of biofilm formation on the surfaces and fissures of dentures. Thus, this study aimed to formulate and evaluate fluconazole tablets for use in preparing a disinfectant mixture with anticandidal activity. For size enlargement of lactose, a tablet diluent, using polyvinylpyrrolidone (PVP) as an agglomerating agent, was developed to enhance the flowability and compactability of the tablet preparation using direct compression. Lactose agglomerates with 6% PVP were used as a diluent for the fluconazole tablets. Furthermore, other excipients were used, such as a buffering agent, disintegrant, surfactant, and lubricant. The fluconazole tablets obtained could be dispersed and dissolved within 10 min in distilled water to achieve a clear mixture, providing a neutral pH and 96% transmittance. Furthermore, the fluconazole mixtures displayed anticandidal efficiency against *C. albicans* with a similar effect to the standard fluconazole solution. These findings suggest that the fluconazole-loaded lactose agglomerate tablets show strong potential when prepared using direct compression. The fluconazole mixtures made by dispersing the tablets can be used as a disinfectant for *Candida*-associated dentures, particularly in patients with oral candidiasis.

## 1. Introduction

Dentures are crucial for helping the chewing system in the absence of teeth. There are complete and partial types of dentures available, depending on the patient’s requirement and convenience. However, wearing dentures long-term can affect the microenvironment of the oral cavity. Irritation or inflammatory responses induced by denture material have led to biofilm formation and microbial colonization [1]. This occurrence has led to the development of denture stomatitis [2]. *Candida* species, particularly *C. albicans*, are one of the leading causes of denture biofilm formation. These fungi adhere to and grow in denture fissures [3]. Moreover, patients with oral candidiasis also have a chance of fungi accumulation on the dentures. Personal hygiene and denture cleaning are essential for relieving denture stomatitis. Commercially available cleaning agents have been shown to prevent the adhesion of *C. albicans* on cell culture plates compared to water [4]. Chlorhexidine, sodium hypochlorite, and hydrogen peroxide have been used as a disinfectant solution on *C. albicans* adhered to acrylic resin dental prosthesis [5]. However, it is difficult to prepare these chemical substances at a concentration suitable for daily use for patients. Therefore, a denture disinfectant tablet with anticandidal activity has been designed and formulated, which can disperse and dissolve in water for soaking dentures. It is intended to eradicate *C. albicans* and promote the oral hygiene of the patient wearing dentures.

Fluconazole is a triazole antifungal drug that disrupts ergosterol synthesis by binding with fungal cytochrome P-450 and subsequently interrupts the functions of fungal membranes [6]. It is active against a broad spectrum of yeast and other fungal pathogens and is available for oral and parenteral uses [7]. Fluconazole has a molecular weight of 306.3 Da. It is a weak base, and its ionization constant (pK_a_) is 1.76 ± 0.10 [8]. The solubility of fluconazole in water has been reported to be 5 mg mL^−1^ [9]. Fluconazole has been used clinically for treating oral candidiasis and is commercially available as a conventional oral capsule (150 mg dose). The minimum inhibitory concentration (MIC) of fluconazole for *C. albicans* has been reported to be in the range of 2–16 µg mL^−1^ [10]. The MIC values of fluconazole against *C. albicans* decreased with increasing the pH of the medium, and the pH has been reported to be in the range 4–7 [11]. This report identified that fluconazole had higher efficiency against *C. albicans* in the neutral pH range. Fluconazole capsules may be used as a disinfectant for dentures for oral candidiasis patients. Fluconazole mixtures are prepared by dispersing powder from capsules in water, and the dentures are immersed into the mixtures overnight to eradicate *C. albicans*. However, the fluconazole mixtures have an undesirable characteristic. The mixture is opaque with high turbidity due to water-insoluble diluents used in the capsules. Therefore, formulating fluconazole tablets as a denture disinfectant with anticandidal activity will be an improvement. The formulated tablets disperse and dissolve to achieve a clear mixture before use, due to the water-soluble properties of fluconazole and the exclusion of water-insoluble excipients.

Tablets as a dosage form have been widely used for a long time because of their ease-of-use and convenient administration, economy, and extended drug stability [12]. This dosage form has compressed drug powder, diluent, and other inactive substances in a limited volume, resulting in a compacted solid form. The direct compression method is a simple process for tablet preparation with low costs and a short timeframe [13]. However, diluents are essential in this preparation method to provide good flowability and compressibility [14,15]. For tablet preparations, lactose, a water-soluble substance, has been widely used as a diluent because of its cost-effectiveness, bland taste, low hygroscopicity, and good physical and chemical stability [16]. However, lactose powder possesses poor flowability [16] and cannot be used for the direct compression method. Therefore, the improvement of lactose powder flowability is required and can be achieved using a size enlargement method. The particle agglomeration method was selected as the approach for increasing the particle size of the powder, which is characterized by adding an agglomerating agent. Many bond types, such as liquid bridges, solid bridges, and interlocking bonds [17], are involved in the agglomeration of powders to promote particle enlargement. Furthermore, this method is not complicated, and the conventional instruments for preparing tablets by wet granulation can be applied. Polyvinylpyrrolidone (PVP) has previously been reported as an agglomerating agent for many polymeric materials [18,19]. The agglomerates obtained possessed better flowability and increased tablet hardness when applying compression pressure.

In this study, the fluconazole tablets were prepared using direct compression. The tablets could disperse and dissolve in water to achieve a clear disinfectant mixture against *C. albicans*. The main diluent used was lactose agglomerate prepared using varying content of PVP as the agglomerating agent. The lactose agglomerates were characterized in terms of particle morphology, flowability, and tablet properties without the drug, which was compared with lactose and spray-dried lactose (SDL). The tablet formulas were composed of fluconazole, lactose agglomerates (diluent), disodium hydrogen phosphate and sodium dihydrogen phosphate (buffering agent), sodium starch glycolate (disintegrant), sodium lauryl sulphate (surfactant), and magnesium stearate (lubricant). The tablets obtained were investigated for thickness, hardness, disintegration time, and tablet dissolution time. In addition, the pH and % transmittance of the mixture prepared using tablets were determined. Moreover, the fluconazole content in the tablets and the anticandidal activity of the fluconazole disinfectant mixtures were also examined.

## 2. Materials and Methods

### 2.1. Materials

Fluconazole was obtained from Hubei YuanCheng Gongchuang Technology Co., Ltd. (Wuhan, China). Lactose in monohydrate and spray-dried lactose monohydrate (SDL, Flowlac^®^100) were purchased from Thai Meochems Co., Ltd. (Bangkok, Thailand). Disodium hydrogen phosphate and sodium dihydrogen phosphate (anhydrous) were obtained from Merck Ltd. (Bangkok, Thailand). In addition, sodium starch glycolate (Explotab^®^, Rama Production Co., Ltd., Bangkok, Thailand), sodium lauryl sulphate (S. Tong Chemicals Co., Ltd., Bangkok, Thailand), and magnesium stearate (S. Tong Chemicals Co., Ltd., Bangkok, Thailand) were employed in this study. *C. albicans* ATCC 10231 was a gift from the Biofilm Research Group, Faculty of Dentistry, Khon Kaen University (Khon Kaen, Thailand). All the other reagents were of analytical grade and used as received.

### 2.2. Preparation of Lactose Agglomerates

PVP in weights of 4, 8, and 12 g was mixed with lactose powder to produce 200 g of the mixture; the content of PVP in the mixtures was 2, 4, and 6% *w*/*w*, respectively. The dry mixtures were mixed using a mortar and pestle until homogeneous. To obtain a wet mass, ethanol (95%) was added to and blended into the mixtures. Then, the wet mass was pressed through a 1.81-mm sieve, dried at 50 °C in a hot air oven, and passed through a 150-μm sieve before particle size selection. The agglomerates in the size range of 75–150 µm were collected using a sieve analyzer. The lactose agglomerates with 2, 4, and 6% *w*/*w* PVP were kept in a desiccator before the test.

### 2.3. Characterization of Lactose Agglomerates

#### 2.3.1. Particle Morphology

The particle morphology of the samples was investigated using scanning electron microscopy (SEM). The samples were mounted on dummies, sputtered with gold in a vacuum evaporator, and then viewed using a scanning electron microscope (Hitachi S-3000N, Tokyo, Japan).

#### 2.3.2. Flow and Compaction Properties

The flowability of lactose, SDL, and the lactose agglomerates was examined using *Carr’s index* and the *Hausner ratio*. The lactose and SDL were investigated for comparison with the lactose agglomerates. The samples (10 g) were weighed and poured into a 50-mL graduated cylinder. The initial volume of the sample was recorded, and the bulk density (*D_B_*) was calculated. Then, the cylinder was tapped 250 times 2 inches from the bottom, and the final volume was used to compute the sample’s tapped density (*D_T_*). Carr’s index (%) and the *Hausner ratio* were computed using the following equations:

$$Carr^{\prime }s\;index=\frac{D_{T}-D_{B}}{D_{T}}\times 100$$

$$Hausner\;ratio=\frac{D_{T}}{D_{B}}$$

The tablets without the drug were prepared using the lactose agglomerates as a diluent. The tablets (250 mg each) comprised 247.5 mg of lactose agglomerates and 2.5 mg of magnesium stearate (1% *w*/*w* of tablet weight) as a lubricant. The lactose agglomerates and magnesium stearate were blended in a rotomixer for 3 min. Then, the 250 mg mixture was weighed and placed into a 10 mm-diameter flat-faced die and punch. A compression pressure of 7.8 MPa was applied using a hydraulic press (Model 3126, Shimadzu, Kyoto, Japan) without holding time. The tablets obtained were stored in a desiccator until investigation. The lactose and SDL tablets were prepared using the same procedure for comparison. The tablets’ thickness and hardness were evaluated using a Vernier caliper and a tablet hardness tester (Model 40-2100 VK200 VanKel^®^, Cary, NC, USA), respectively. Furthermore, the surface morphology of tablets was also examined using SEM.

### 2.4. Formulation and Preparation of Fluconazole Tablets

The formulas of the fluconazole tablets developed in this study are listed in Table 1. Fluconazole was the active ingredient in these tablets with a content of 20% *w*/*w*. The buffering agents used were disodium hydrogen phosphate and sodium dihydrogen phosphate in various ratios, with 15% *w*/*w* content. The lactose agglomerates were employed as a diluent. Sodium starch glycolate, magnesium stearate, and sodium lauryl sulphate were used as the disintegrant, lubricant, and surfactant, respectively.

Each tablet (300 mg) contained 60 mg of fluconazole and the other components. Fluconazole and the other components were blended using a geometric dilution in a rotomixer for 5 min. Finally, magnesium stearate was added to the mixtures and mixed for 3 min. The mixture (300 mg) was weighed, and a 10 mm-diameter flat-faced die and punch was filled. A compression pressure of 7.8 MPa was applied using a hydraulic press (Model 3126, Shimadzu, Kyoto, Japan) without holding time. The fluconazole tablets were kept in a desiccator before characterization.

### 2.5. Evaluation of Fluconazole Tablets

The thickness and hardness of the tablets were evaluated using the apparatuses mentioned in the ‘flow and compaction properties’ section. The tablet disintegration time was determined by a disintegration tester (Model ZT-324, Erweka America Inc., Edison, NJ, USA). It consisted of a basket–rack assembly in a 1000-mL beaker filled with 750 mL of distilled water at 37.0 ± 0.5 °C. The tablet was placed into the basket without a dish, and the baskets were vertically moved. The disintegration time was recorded when the tablet was liberated entirely from the basket screen.

Tablet dissolution time was tested in this study. The 500-mL glass beaker was filled with 300 mL of distilled water at room temperature (26–28 °C). A three-blade overhead stirrer (WiseStir^®^ HS-100D, Daihan Scientific Co., Ltd., Gangwon, Korea) was set in the beaker, and the distance between the lower edge of the blade and the inside bottom of the beaker was maintained at 3 cm during the test. The tablet was placed into the beaker, and the rotation speed of the stirrer was 300 revolutions min^−1^. The tablet dissolution time was noted when the tablet was wholly dispersed from the bottom of the beaker. After that, the pH of the fluconazole mixture was measured using a pH meter (Walklab, Trans Instruments (Singapore) Ltd., Singapore). The clarity of the fluconazole mixture was expressed as a % transmittance, which was measured using a UV-visible spectrophotometer (Model UV1201 Shimadzu, Kyoto, Japan). The sample filled a 1-cm cell, and % transmittance was measured at the wavelength of 500 nm. Distilled water was used as a reference for 100% transmittance.

The fluconazole content in the tablets was determined using HPLC. The tablet was placed into a 50-mL volumetric flash that contained an acetonitrile–water solvent in the ratio of 3:7. The volumetric flash contained a tablet, and the solvent was sonicated for 30 min for a complete dissolution of fluconazole. The supernatant was filtered using a 0.2-µm nylon syringe filter. The filtrate was diluted 25-fold with the acetonitrile–water solvent before analysis by HPLC.

### 2.6. HPLC Analysis

The concentration of fluconazole in the test solutions was analyzed using an HPLC system that consisted of Waters 1525 binary pumps, 2489 dual absorbance detector, and Waters Breeze Software 2.0 versions (Waters Corporation, Milford, MA, USA). A C-18 reversed-phase HPLC column (4.6 mm × 150 mm) with a 5-μm particle size packing material (Mightysil RP-18 GP, Kanto Chemical Co., Inc., Tokyo, Japan) was used. The mobile phase was acetonitrile:water (30:70) with a flow rate of 1 mL min^−1^. The detection wavelength was set at 210 nm. The retention time of fluconazole was approximately 2.8 min. Under these conditions, good linearity and reproducibility were demonstrated over the 20–60 μg mL^−1^ fluconazole range. The limit of detection and limit of quantification, which were determined and computed using a linear regression curve [20], were found to be 18.22 and 55.21 µg mL^−1^, respectively. Additionally, the relative standard deviations of the intra-day and inter-day variations of the fluconazole peak area at the concentrations of 20 and 40 µg mL^−1^ were less than 1 and 2%, respectively.

### 2.7. Anticandidal Activity Studies

Fluconazole tablets (60 mg each) were dispersed and dissolved in 300 mL distilled water to achieve the mixtures. The mixtures prepared from F5 and F6 tablets were tested for anticandidal activity against *C. albicans* using an agar diffusion method. This method has been used to evaluate drug formulations’ antifungal activity [21,22,23]. First, *C. albicans* was inoculated into 20 mL of a Sabouraud dextrose broth. At the exponential period of growth, the culture broth was diluted. The concentration of *C. albicans* was diluted to 106 CFU mL^−1^. Then, plates containing 20 mL of Sabouraud dextrose agar were prepared, in which 200 μL of the microbe suspension was added and spread uniformly with sterile cotton swabs. The agar was punched using a Pasteur pipet to create 6-mm-diameter holes, and four holes were produced in each plate. The 45 μL solutions of the negative control, positive control, and test mixtures were dropped into the holes. All plates were incubated at 37.0 ± 0.5 °C for 24 h. Next, the diameters of the inhibition zones were measured. The negative control was distilled water, and the positive control was 200 mg mL^−1^ of standard fluconazole solution.

## 3. Results

### 3.1. Characteristics of Lactose Agglomerates

**Figure 1:**
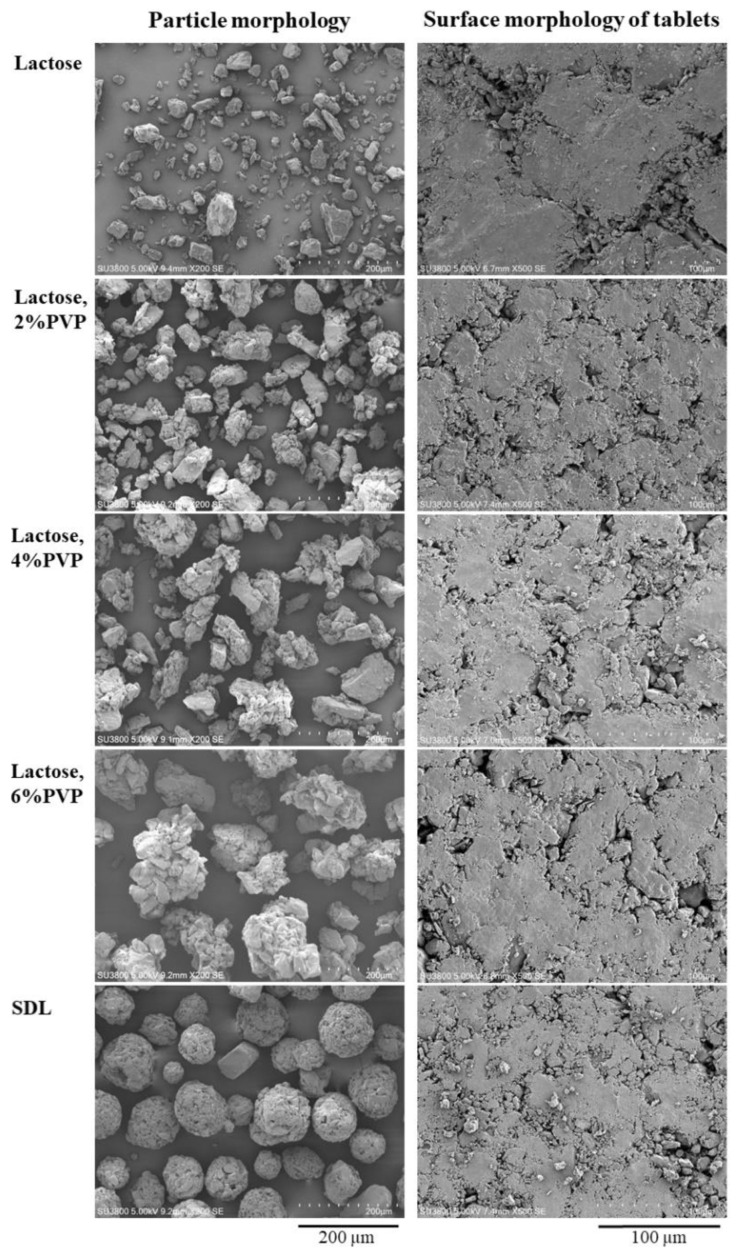
Particle morphology of lactose, SDL, and lactose agglomerates and surface morphology of tablets prepared using various diluents.

The particle morphologies of lactose, SDL, and lactose agglomerates viewed using SEM are shown in Figure 1 (left panel). An irregular shape of lactose particles and lactose agglomerates was observed, whereas SDL presented spherical particles. The lactose agglomerates had a bigger particle size than lactose, and increasing the PVP content caused an increase in the size of the lactose agglomerates. It was observed that the PVP in the PVP–lactose mixtures was dissolved when 95% ethanol was added during preparation. The PVP dissolved to create a thin film (liquid bridge in the wet state) and induced adhesion of the lactose particles, leading to the formation of capillary forces and strong solid bridges between particles [17,24].

The characteristics of the lactose agglomerates are listed in Table 1. The bulk density values of lactose and the lactose agglomerates were in the range of 0.39–0.41 g cm^−3^, whereas the lactose agglomerates presented lower tapped density values than lactose. These results led to the highest Carr’s index and the Hausner ratio of lactose, and the flowability was very poor. The particle agglomeration of lactose enhanced the flowability by decreasing the values of Carr’s index and the Hausner ratio. An increase in PVP contents resulted in a continuous decrease in both parameters. The lactose agglomerates with 6% PVP displayed a good level of flowability. Compared with SDL, the lactose agglomerates had lower bulk and tapped densities. This result may be due to the different particle shapes of both diluents. SDL had a spherical shape, presented by a close-packed arrangement of particles, leading to higher bulk and tapped densities when compared with the irregular shape of the lactose agglomerates. The flow property of SDL was passable in this investigation condition.

The 250 mg tablets without drug using the diluents were compressed at 7.8 MPa, and the tablet characteristics are shown in Table 1. The thickness of the tablets was in the range of 2.47–1.60 mm. The hardness of the lactose and SDL tablets was 16.87 ± 0.78 and 22.58 ± 2.03 N, respectively, suggesting that SDL demonstrated better compactability than lactose. On the other hand, the lactose agglomerate tablets showed remarkably higher hardness when compared with the lactose and SDL tablets. The hardness of the lactose agglomerate tablets increased with increasing % PVP content. The surface morphology of the tablets was viewed using SEM, as shown in Figure 1 (right panel). The lactose, SDL, and lactose agglomerates particles were cracked and compacted under pressure. It is well known that the main deformation of lactose is the particle fragmentation under compression [14,16]. The large surface area of the small fragments formed a bond between particles. However, Ilić et al. (2009) reported the plasticity of lactose materials under compression pressure and showed that SDL possessed higher plasticity than lactose. This may explain why the SDL obtained greater tablet hardness than lactose. Furthermore, the results in this study also suggested that PVP added enhanced compatibility to the tablets by increasing cold welding (strong bonding) between particles [25], leading to the enhancement of the tablet hardness. A similar effect on the tablet hardness of polymeric materials has also been reported previously [19]. Thus, the lactose agglomerates with 6% PVP showed good flowability and compatibility and were selected to be used in the formulation of the fluconazole tablets.

### 3.2. Effect of Buffering Agent on Tablets

The formulas of the fluconazole tablets are shown in Table 2. All the formulations contained 20% fluconazole. The buffering agents used in this study were disodium hydrogen phosphate and sodium dihydrogen phosphate, which made up 15% of the tablet content. The buffering agents were used in the anhydrous form, because the hydrate caused a eutectic mixture in mixing and tableting during the tablet preparation process. Boric acid, a water-soluble substance, was used as a lubricant for tableting. However, the compressed tablets were difficult to eject out of the die after tableting due to the tablet sticking to the die and punch. Magnesium stearate, a water-insoluble substance, provided better efficiency in the tablet ejection process in this study. Therefore, 1% *w*/*w* of magnesium stearate was employed as a lubricant.

**Figure 2:**
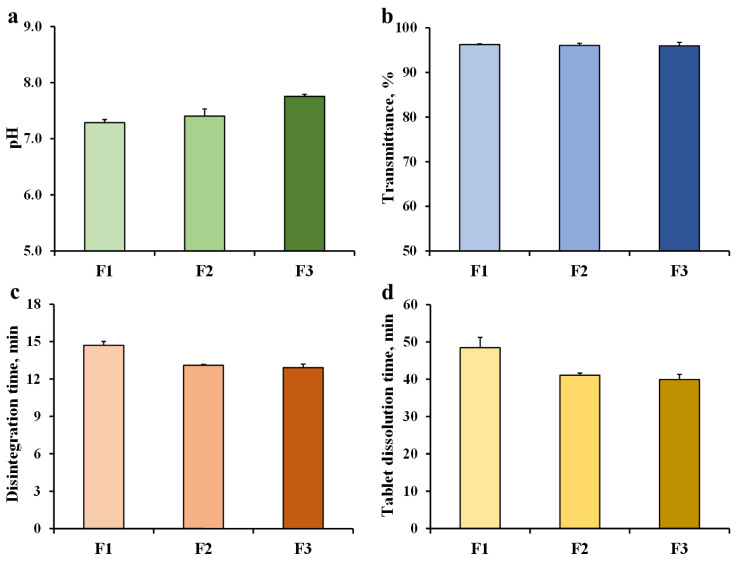
Effect of buffering agents on pH (**a**) and % transmittance (**b**) of fluconazole mixtures, disintegration time (**c**), and tablet dissolution time (**d**) of fluconazole tablets. Each value is the mean ± S.D., n = 3.

F1, F2, and F3 used different ratios of disodium hydrogen phosphate and sodium dihydrogen phosphate with exactly 15% *w*/*w* content. The fluconazole tablets (250 mg) of the F1–F3 formulations were prepared and dissolved in 300 mL-distilled water. The pH of the fluconazole mixtures of the F1–F3 tablets are presented in Figure 2a. The pH of the mixtures increased with the disodium hydrogen phosphate ratio, which acted as a salt in the buffer system. Therefore, the pH of the F1–F3 mixtures was suitable for the anticandidal activity of fluconazole. The neutral pH showed a lower MIC for *C. albicans* [11]. The transmittance values of the F1, F2, and F3 mixtures were in the range of 95–96% when compared with the distilled water (Figure 2b). A decrease in % transmittance of the mixture was due to a water-insoluble substance, such as 1% *w*/*w* magnesium stearate, used in tablets.

The disintegration time of the F1 tablets was 14.7 min, and increasing the disodium hydrogen phosphate ratio resulted in a decrease in the disintegration time (Figure 2c). This result may have been caused by the decreased tablet hardness when disodium hydrogen phosphate was increased. The F3 tablets demonstrated the lowest tablet hardness (Table 2). The results of the disintegration time affected the time for complete dissolution of the tablets, as shown in Figure 2d. The F1 tablets demonstrated the longest dissolution time (48.5 min), whereas the slowest dissolution time (39.9 min) was demonstrated by the F3 tablets. The F1, F2, and F3 tablets were disintegrated in distilled water using the dissolution process, which was the main mechanism, because almost all tablet components were able to be dissolved in water. However, the disintegrant was not added in these formulations. A faster disintegration time was obtained compared to the tablet dissolution time because the baskets that contained the tablets in the disintegration apparatus moved vertically to apply mechanical stress to the tablets, which accelerated the disintegration of the tablets.

### 3.3. Effect of Disintegrant on Tablets

**Figure 3:**
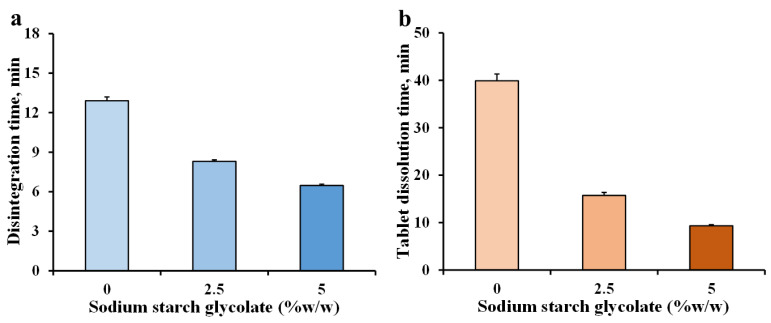
Effect of sodium starch glycolate on disintegration time (**a**) and tablet dissolution time (**b**) of fluconazole tablets. Each value is the mean ± S.D., n = 3.

The fluconazole tablets should be left for a short time in water for dispersing and dissolving to achieve a mixture. The F3 sample was selected to incorporate the disintegrant to accelerate the disintegration and tablet dissolution times. Sodium starch glycolate, a superdisintegrant [26,27], was used in this study. The F3, F4, and F5 samples contained different sodium starch glycolate amounts of 0, 2.5, and 5% *w*/*w*, respectively (Table 2). The weight of the tablets was in the range of 296–298 mg, and the higher the sodium starch glycolate content was, the lower the tablet thickness (Table 2). Moreover, the hardness increased with the increasing content of sodium starch glycolate. This result may have been due to the plastic deformation of the starches, which can enhance the cold welding between particles. The pH of the F3, F4, and F5 fluconazole mixtures was 7.76 ± 0.04, 7.82 ± 0.03, and 7.52 ± 0.01 (n = 3), respectively, suggesting that this parameter was decreased by adding 5% *w*/*w* of sodium starch glycolate. In contrast, the mean transmittance of the solutions was found to be 95.9, 95.2, and 96.0% (n = 3), respectively. This result showed indifference to the transmittance of the mixtures. The added disintegrant influenced the times of disintegration and tablet dissolution remarkably, as shown in Figure 3. The addition of 5% *w*/*w* sodium starch glycolate reduced the disintegration time two-fold and 4.3-fold for the tablet disintegration time, even though the hardness of the F5 tablets was increased (Table 2). It has been shown that the sodium starch glycolate swells when exposed to water, leading to increased pressure within the tablet structure, which overcomes the intermolecular bonds between the component particles, leading to tablet disintegration [26]. This phenomenon led to shorter times for disintegration and tablet dissolution in this study.

The disintegration study of the F5 tablets identified a short disintegration time for this formulation. Regarding drug dissolution, it was not essential to test the fluconazole dissolution in this study, because fluconazole is slightly soluble in water; the solubility in water is 5 mg mL^−1^. Thus, the formulated 60-mg-fluconazole tablets can be rapidly dissolved in 300 mL of water after tablet disintegration. Moreover, an oral fluconazole immediate-released tablet demonstrates very rapid dissolution in various pHs of dissolution media; therefore, a biowaiver was recommended for this dosage form [28].

**Figure 4:**
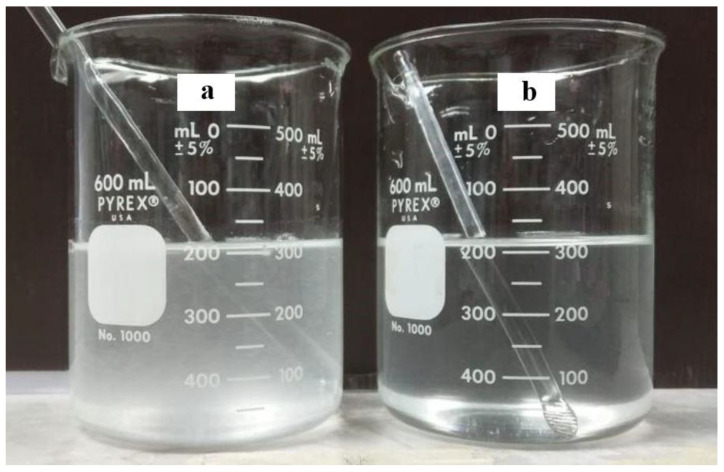
Appearance of the mixtures prepared using the 150-mg -fluconazole capsule (**a**) and F5 tablet (**b**) in 300 mL of distilled water.

For the visual inspection of the F5 mixture, the F5 tablets, when dispersing and dissolving in 300 mL of water, presented quite a clear mixture, as illustrated in Figure 4. In contrast, the 150 mg fluconazole capsules dispersed in 300 mL of water resulted in a higher turbidity mixture when compared to the F5 mixture. Thus, a better appearance was obtained by the F5 mixture.

### 3.4. Effect of Surfactant on Tablets

**Figure 5:**
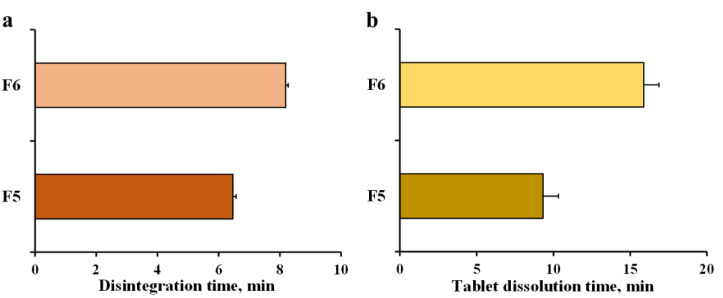
Disintegration time (**a**) and tablet dissolution time (**b**) of fluconazole tablets without (F5) and with (F6) sodium lauryl sulphate. Each value is the mean ± S.D., n = 3.

Sodium lauryl sulphate was used as a surfactant at 10% *w*/*w* of the content of the formulation. Surfactants reduce the surface tension of water, helping it to wet and spread out more uniformly. In addition, the surface free energy on a denture surface is related to the adhesion of micro-organisms, such as a *Candida* colonization [2]. This effect can be reduced by adding a surfactant, which decreases the surface free energy of materials, resulting in the easy wetting of the denture surface. Thus, it could be expected that sodium lauryl sulphate may enhance the anticandidal activity of fluconazole solution. F6 tablets were used as the formulation with added 10% *w*/*w* sodium lauryl sulphate, and the characteristics of the fluconazole tablet and solution were compared to those of the F5 tablets. The weight of the F5 and F6 tablets were comparable, but the thickness of the F6 tablets was higher than that of the F5 tablets (Table 2). In contrast, the F6 tablets demonstrated a lower level of hardness. These results were found by decreasing the lactose agglomerate content in the F6 tablets when sodium lauryl sulphate was incorporated. The pH of fluconazole mixtures prepared using F5 and F6 tablets was 7.52 ± 0.01 and 7.33 ± 0.02, respectively. The sodium lauryl sulphate caused a reduction in the pH of the fluconazole mixture. However, the transmittance of both mixtures was approximately 96% because of the water-soluble property of sodium lauryl sulphate. Although sodium lauryl sulphate decreased the hardness of the tablets, a longer disintegration time and tablet dissolution time were obtained (Figure 5). This result suggests that sodium lauryl sulphate might influence the swelling of the disintegrants, and the swelling pressure of the disintegrants might decrease the hardness of tablets.

### 3.5. Anticandidal Activity of Fluconazole Mixtures

The anticandidal activity of the fluconazole mixtures prepared from the F5 and F6 tablets was tested using an agar diffusion method. This method was selected because it is one of the standard methods for susceptibility testing in routine laboratory testing. It is easy to handle and economical. However, it is impossible to quantify the drug amount diffused into the agar medium [29]. Any remaining microcolonies of *C. albicans* in the inhibition zone cannot be visibly observed. Furthermore, it is dependent on stringent standardization of the inoculum [30]. However, this method was used in this study for anticandidal activity comparison between the fluconazole mixtures prepared from the tablets developed and standard fluconazole solution.

**Figure 6:**
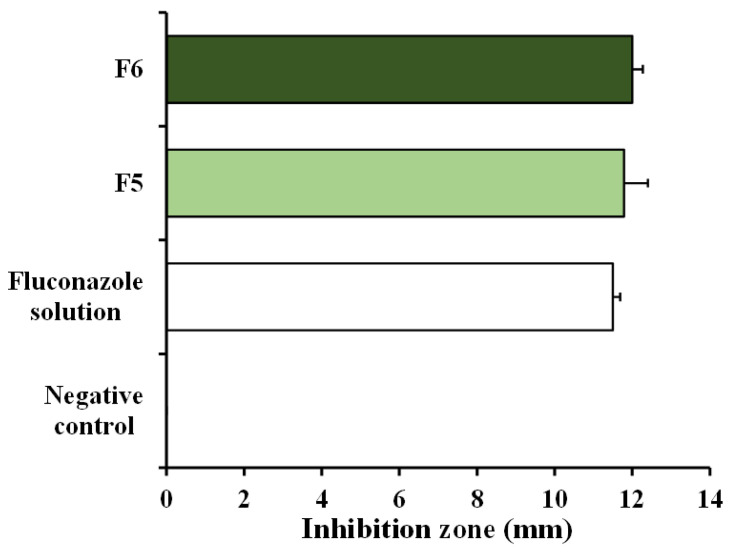
Anticandidal activity expressed as the inhibition zone of the fluconazole mixtures prepared from F5 and F6 tablets. Each value is the mean ± S.D., n = 3.

Before this test, the tablet fluconazole content was analyzed using HPLC. The fluconazole content in the F5 and F6 tablets was determined to be 59.40 ± 1.82 and 59.37 ± 4.74 mg/tablet (n = 3), respectively. This was calculated as the % label amount of 99.00 ± 3.03 and 98.95 ± 7.90%, respectively. The inhibition zones of the F5 and F6 mixtures are displayed in Figure 6. The F5 and F6 mixtures at 200 µg mL^−1^ fluconazole provided a similar inhibition zone to the standard fluconazole solution (positive control) at the same concentration. This result indicates that the tablet excipient and preparation process used did not affect fluconazole’s efficiency against *C. albicans* compared to the standard fluconazole solution.

## 4. Conclusions

Fluconazole tablets were successfully prepared using lactose agglomerates as a diluent. The lactose agglomerates prepared using 6% PVP provided good flowability and compactability for tablet preparation using the direct compression method. The tablet formulation was composed of a buffering agent with disodium hydrogen phosphate and sodium dihydrogen phosphate, sodium starch glycolate, sodium lauryl sulphate (if necessary), and magnesium stearate. The fluconazole tablets obtained could be dispersed and dissolved within 10 min in distilled water to achieve quite a clear mixture. Fluconazole in the concentration of 200 µg mL^−1^ in the mixture displayed similar anticandidal activity when compared with the standard fluconazole solution. This study suggests that the fluconazole-loaded lactose agglomerate tablets present strong potential when prepared using direct compression. Furthermore, the fluconazole mixtures obtained from dispersing and dissolving the tablets can be employed as a disinfectant for *Candida*-associated dentures and also for the dentures of patients with oral candidiasis.

## Figures and Tables

**Table 1:** Characteristics of lactose agglomerates in the forms of powders and tablets.

Characteristics	Lactose	Lactose Agglomerates			SDL
		2% PVP	4% PVP	6% PVP	
Powders ^a^					
-Bulk density	0.40 ± 0.01	0.39 ± 0.01	0.40 ± 0.01	0.41 ± 0.01	0.58 ± 0.01
-Tapped density	0.61 ± 0.01	0.50 ± 0.01	0.50 ± 0.01	0.47 ± 0.01	0.71 ± 0.01
-Carr’s index	34.67 ± 1.15	21.86 ± 0.49	19.15 ± 0.96	14.17 ± 1.76	22.36 ± 1.44
-Hausner ratio	1.53 ± 0.03	1.27 ± 0.01	1.24 ± 0.01	1.17 ± 0.02	1.20 ± 0.02
-Flow property	Very poor	Passable	Fair	Good	Passable
Tablets ^b^					
-Weight (mg)	247.44 ± 0.47	248.32 ± 0.61	249.32 ± 0.80	248.70 ± 0.45	247.92 ± 0.42
-Thickness (mm)	2.50 ± 0.02	2.47 ± 0.04	2.51 ± 0.02	2.60 ± 0.02	2.53 ± 0.02
-Hardness (N)	16.87 ± 0.78	43.64 ± 6.47	46.09 ± 5.59	62.76 ± 5.98	22.58 ± 2.03

^a^ Data are mean ± S.D., n = 3; ^b^ data are mean ± S.D., n = 5.

**Table 2:** Formulations and characteristics of fluconazole tablets.

Component	Content in Formulation (% *w*/*w*)					
	F1	F2	F3	F4	F5	F6
Fluconazole	20.0	20.0	20.0	20.0	20.0	20.0
Disodium hydrogen phosphate	10.0	11.7	13.3	13.3	13.3	13.3
Sodium dihydrogen phosphate	5.0	3.3	1.7	1.7	1.7	1.7
Lactose agglomerates	64.0	64.0	64.0	61.5	59.0	49.0
Magnesium stearate	1.0	1.0	1.0	1.0	1.0	1.0
Sodium starch glycolate	-	-	-	2.5	5.0	5.0
Sodium lauryl sulphate	-	-	-	-	-	10.0
**Tablet Characteristics**	**F1**	**F2**	**F3**	**F4**	**F5**	**F6**
Weight (mg)	299.1 ± 0.7	298.6 ± 0.8	296.4 ± 0.8	297.6 ± 1.5	298.26 ± 0.6	299.1 ± 0.5
Thickness (mm)	2.94 ± 0.03	2.89 ± 0.02	2.89 ± 0.03	2.89 ± 0.03	2.78 ± 0.01	2.96 ± 0.04
Hardness (N)	71.98 ± 2.82	74.73 ± 4.07	58.25 ± 4.36	60.41 ± 1.64	79.63 ± 3.63	64.53 ± 4.57

Data of tablet characteristics are mean ± S.D., n = 5.

## Data Availability

Data are contained within the article.
